# Codesign and system integration as interacting demands in digital health for youth experiencing chronic pain

**DOI:** 10.1097/PR9.0000000000001494

**Published:** 2026-09-01

**Authors:** Niranjan Bidargaddi, Tammie R. Foster, Andrew M. Briggs, Samantha Rowbotham, Helen Slater

**Affiliations:** a Digital Health Research Lab, Flinders Health and Medical Research Institute, Flinders University, Adelaide, South Australia, Australia; b Curtin School of Allied Health, Curtin University, Perth, Western Australia, Australia

## Abstract

Codesign and system integration should be treated as a single, interacting design requirement in moving digital youth pain health solutions towards equitable, sustained, embedded care.

## 1. The unmet need

Chronic musculoskeletal pain (CMP) affects about one in five young adults worldwide,^6,25^ disrupting young people's education, employment, social participation, and overall wellbeing.^6,20,24^ Here, CMP refers to pain persisting or recurring for more than 3 months in musculoskeletal structures, including primary and secondary pain conditions.^36^ We focus on this population because musculoskeletal pain is one of the most prevalent conditions experienced by young people aged 16 to 24 years and is a leading driver of participation restriction, functional disability, and clinical need.^32^ Young people in this age group experience stigma and self-doubt around CMP, with resulting disengagement from care,^20,21,30^ and the cumulative effect is an escalating disease burden and persisting care gap between need and service provision at a critical life stage.^27^ Although many of the design and integration principles discussed here may apply to other ICD-11 chronic pain classifications,^36^ their service contexts and evidence base differ and are not addressed here.

## 2. How current systems fall short

Traditional pain service models seldom accommodate the dynamic, transitional nature of moving from adolescence into young adulthood, with inadequately integrated pain care, including for mental health comorbidity.^20,21^ Clinicians often lack time, resources, and skills to provide biopsychosocial, holistic person-centred pain care. Young people often turn to informal online sources to seek advice or may disengage from formal care entirely,^5^ feeling left behind in the health system, lost between service boundaries, and uncertain where to seek supportive care.^20,35^

Digital health solutions such as health applications (“apps”), telehealth, and wearable devices offer promise for helping to address care inequity by improving access and extending care support, reaching across geographic and institutional boundaries.^3,23^ However, these solutions are often developed in isolation from the health systems in which they are expected to operate. Solution integration into care delivery is typically retrospective to development. Consequently, many digital solutions score well on user experience but poorly on system integration.^7,29^ This raises the question of how to bring system-level and end-user requirements into codesign from the outset.

## 3. Codesign and system integration as a single design problem

Despite supportive national digital health strategies,^1,11,12^ regulatory and operational frameworks frequently lag, constraining implementation of novel solutions for young people's pain care.^4^ At the patient level, digital solutions must be engaging, accessible, equitable and developmentally appropriate, fitting young people's routines, preferences, literacy levels,^8^ and risk perceptions.^9^ For clinicians, solutions must align with clinical workflows, be a value-add, satisfy clinician/patient relationship standards, and comply with governance and regulatory frameworks.^28^ Usability is essential for adoption, but end-user preferences, confidence, and digital literacy also determine whether solutions are trusted and embedded in clinical care.^4,8,17,28^ At the organisational level, health care services must also accommodate digital innovation without undermining clinical time, quality, or safety.^37^ At the system level, supportive policy, governance, and aligned funding models are required to support innovation and sustain implementation.^2^

Codesign is widely promoted as a way to make digital health solutions “fit for purpose” across multiple health system levels.^34,38,40^ Involving young people from the outset in shaping content, tone, and features strengthens relevance, trust, and engagement.^13,30,35,40,41^ Codesigned interventions such as iCanCope with Pain, WebMAP, and SOLACE projects demonstrate improved adherence and real-world usability,^13,22,31,33^ whereas ECHO Pain project applies a related approach to workforce capacity building to support pain care delivery through expert clinical telementoring.^10,15,18^ These initiatives have each strengthened important parts of the pain care ecosystem.^29^

A recalcitrant issue remains the failure in codesign to consider organisational workflows, referral pathways, governance arrangements, and funding mechanisms, which are often treated as integration challenges to be solved after the solution is built. Service managers, finance leads, IT, and governance staff are routinely involved in digital solution decisions, but mainly through procurement and deployment, assessing near-finished products against the service's capacity to absorb them into routine practice. Prior work has highlighted limited payer and service-side stakeholders involvement in digital health codesign^19,39^ and examined procurement of digital health solutions as an integration challenge in its own right.^14^ Service-level integration is usually considered only after design is complete, treating solutions as fixed and positioning service constraints as late-stage acceptance criteria, rather than design inputs. Stakeholders with expertise in operational alignment are engaged through misaligned forums, so critical requirements remain invisible until postdevelopment. Bringing system- and service-level requirements inside the codesign process from the outset means engaging these stakeholders as genuine codesign partners, and treating their constraints as inputs informing what the solution becomes.

## 4. A worked example: myPAinhealTH

To illustrate this approach, we outline the myPAinhealTH (myPATH) digital health program, currently under development in Australia. In this program, from inception, the system manager and the clinical service have been codesign partners, alongside end users (young people and clinicians), and concurrently with authorities who can commit to operational change (service managers, business and finance, IT, governance leads). Running these streams in parallel allows design choices in each, to adapt to constraints emerging in the others. An example from the current design phase illustrates the point. A preference for an AI chat feature from young people surfaced an immediate workflow constraint. Any such feature would generate safety alerts that the clinical service has no capacity to absorb. Without service-side codesign, this constraint would not have surfaced until after development, the feature would likely have been built and then dropped. Because the clinical service was engaged from the outset, a safety response layer was created outside the existing workflow to handle alerts, with operational protocols extended to enable information-sharing and agreed response timeframes. Whether this design delivers sustained value remains to be established; however, this kind of operational extension does not surface when codesign is confined to end users alone.

## 5. Future directions and policy implications

Treating codesign and system integration as a single design problem has direct implications for how digital pain care for young people is developed, implemented, funded, and evaluated. At the funding level, schemes supporting digital health development should require, at the proposal stage, evidence that organisational workflows and governance stakeholders are engaged in the codesign process from inception. Most current digital health pilot funding does not do this, effectively subsidising the pattern we identify as problematic. Although Australia's National Digital Health Strategy Roadmap recognises the need to prioritise integrated digital health solutions,^2^ operationalising this priority means changing what funders require. Specific criteria are critical to mandate early and sustained involvement of organisational workflows and governance stakeholders in codesign at the funding application stage. At the service organisation level, clinicians, service managers, and governance leads need to participate in codesign as design partners. The time required for this participation needs to be funded in the development budget. Implementation research can then guide how the resultant solutions can embed within care pathways, supporting rapid triage, early screening, targeted self-management, and primary-specialist co-care.^28,29^

Evaluation frameworks need a corresponding shift, beyond assessing effectiveness as a single outcome at a defined timepoint. Assessing whether young people and clinicians were meaningfully consulted in codesign is no longer sufficient.^26^ Evaluations should also assess whether organisational workflows, referral pathways, and governance arrangements were genuinely inside the codesign process, whether the resulting solution is embedded in care or sits alongside services and care pathways, what benefits were observed, and related scaling and sustainability implications. Future evaluation frameworks should incorporate goals defined by young people, such as quality of life, engagement, and equity impact,^8,28,37^ alongside services and system-embedding criteria that capture whether the integration intent faithfully delivers on the original value proposition. Meaningful engagement of young people must extend from design to governance, involving young people in oversight and evaluation preserves relevance, trust, equity, and inclusion.

Generic codesign methods can also underserve those from diverse backgrounds, where pain experiences and access barriers are shaped by factors that mainstream codesign processes often fail to surface (eg, education, literacy, cultural backgrounds, language barriers, gender imbalances).^16^ Addressing this consideration requires deliberate methodological adaptation and longer engagement timelines, along with evaluation to ensure marginalised voices are captured. Robust governance and equitable access to digital infrastructure remain essential to realise a connected, trustworthy, and future-ready pain care ecosystem. We acknowledge this as a limitation of most existing initiatives, including those discussed here.

## 6. Conclusions

Young people with CMP face significant barriers to effective, developmentally appropriate pain care. Digital health solutions have multiplied, yet most have been built with users inside the design process and with services and systems partners left outside. The shift this perspective proposes is scoping codesign from inception to include the people with authority over workflows, pathways, governance, and funding, alongside young people and clinicians, so that integration is part of the design brief, not a downstream problem. Sustained system-level commitment will be essential for building a coordinated, connected young people-centered pain care ecosystem.

## Disclosures

The authors of this manuscript (H.S., A.M.B., N.B.) are Chief Investigators on the myPAinHealth grant. S.R. is the Senior Research Manager for this research program. N.B. is a cofounder and shareholder of CareMappr Pty Ltd, building the myPATH digital solution. T.F. declares no competing interests related to this manuscript. The conceptual argument advanced in this perspective does not depend on the success or scale-up of any specific initiative, including myPATH.

## References

[1] Australasian Institute of Digital Health. AIDH strategic plan 2021-2025: Healthier lives, digitally enabled, 2020. Available at: https://digitalhealth.org.au/wp-content/uploads/2021/04/AIDH-Strategic-Plan-2021-25_April-2021.pdf. Accessed September 8, 2025.

[2] Australian Digital Health Agency. National digital health strategy roadmap 2023-2028, 2023. Available at: https://www.digitalhealth.gov.au/sites/default/files/documents/national-digital-health-strategy-roadmap-2023-2028.pdf. Accessed September 12, 2025.

[3] Australian Institute of Health and Welfare. Digital health. Australian Institute of Health and Welfare, 2024. Available at: https://www.aihw.gov.au/reports/australias-health/digital-health. Accessed January 28, 2026.

[4] Borges do Nascimento IJ Abdulazeem H Vasanthan LT Martinez EZ Zucoloto ML Østengaard L Azzopardi-Muscat N Zapata T Novillo-Ortiz D. Barriers and facilitators to utilizing digital health technologies by healthcare professionals. NPJ Digit Med 2023;6:161. 37723240 10.1038/s41746-023-00899-4 PMC10507089

[5] Brown CL Restall G Diaz FAS Anang P Gerhold K Pylypjuk H Wittmeier K. Understand me: youth with chronic pain on how knowledge gaps influence their pain experience. Can J Pain 2023;7:2146489. 36733474 10.1080/24740527.2022.2146489 PMC9888456

[6] Chambers CT Dol J Tutelman PR Langley CL Parker JA Cormier BT Macfarlane GJ Jones GT Chapman D Proudfoot N Grant A Marianayagam J. The prevalence of chronic pain in children and adolescents: a systematic review update and meta-analysis. PAIN 2024;165:2215–34. 38743558 10.1097/j.pain.0000000000003267 PMC11404345

[7] Cherkin DC. How can the intractable problem of chronic musculoskeletal pain (CMP) be effectively managed? The need for a well-integrated systems approach. J Gen Int Med 2018;33(suppl 1):S4–6. 10.1007/s11606-018-4362-5 PMC5902351 29633132

[8] Chua J Slater H Rowbotham S Klem N-R Lord SM O'Sullivan PB Tory B Smith AJ Stinson JN Hansen P Briggs AM. What informs the choices young people living with chronic musculoskeletal pain make about their care? A qualitative analysis of focus groups with young people in Australia. Disabil Rehabil 2025;48:3727–47. 41252593 10.1080/09638288.2025.2585762

[9] Chua J Slater H Rowbotham S Hansen P Smith AJ Lord SM O'Sullivan PB Tory B Stinson JN Briggs AM. What are the valued attributes and perceived risks of harm in digital technologies and AI-enabled digital coaches among youth living with chronic musculoskeletal pain? An exploratory, mixed-methods study. J Pain 2026;43:106257. 41825515 10.1016/j.jpain.2026.106257

[10] De Morgan S Walker P Blyth FM Daly A Burke ALJ Nicholas MK. A technology-enabled collaborative learning model (Project ECHO) to upskill primary care providers in best practice pain care. Aust J Prim Health 2024;30:PY24035. 39699998 10.1071/PY24035

[11] Department of Health and Social Care, NHS England. A plan for digital health and social care, 2022. Available at: https://www.gov.uk/government/publications/a-plan-for-digital-health-and-social-care/a-plan-for-digital-health-and-social-care. Accessed September 8, 2025.

[12] Digital Health Canada. Digital health Canada strategic plan. Digital Health Canada, 2025. Available at: https://digitalhealthcanada.com/about/strategy/. Accessed September 8, 2025.

[13] Elbers S van Gessel C Renes RJ van der Lugt R Wittink H Hermsen S. Innovation in pain rehabilitation using co-design methods during the development of a relapse prevention intervention: case study. J Med Int Res 2021;23:e18462. 10.2196/18462 PMC7857944 33470937

[14] Holterman S Hettinga M Buskens E Lahr M. Factors influencing procurement of digital healthcare: a case study in Dutch district nursing. Int J Health Pol Manag 2022;11:1883–93. 10.34172/ijhpm.2021.115 PMC9808215 34634888

[15] iCanCope Crew. A new virtual research opportunity for youth with chronic pain. Paediatric Project ECHO, 2026. Available at: https://sickkids.echoontario.ca/icancopecrew/. Accessed January 13, 2026.

[16] Jiang Q Naseem M Lai J Toyama K Papalambros P. Understanding power differentials and cultural differences in Co-design with marginalized populations. Proceedings of the 5th ACM SIGCAS/SIGCHI conference on computing and sustainable societies, 2022; 165–79. doi: 10.1145/3530190.3534819.

[17] Jones C Thornton J Wyatt JC. Enhancing trust in clinical decision support systems: a framework for developers. BMJ Health Care Inform 2021;28:e100247. 10.1136/bmjhci-2020-100247 PMC8183267 34088721

[18] Katzman JG Comerci G Boyle JF Duhigg D Shelley B Olivas C Daitz B Carroll C Som D Monette R Kalishman S Arora S. Innovative telementoring for pain management: project ECHO pain. J Continu Educ Health Profess 2014;34:68–75. 10.1002/chp.21210 24648365

[19] Kilfoy A Hsu T-CC Stockton-Powdrell C Whelan P Chu CH Jibb L. An umbrella review on how digital health intervention co-design is conducted and described. Npj Digit Med 2024;7:374. 39715947 10.1038/s41746-024-01385-1 PMC11666587

[20] Klem N-R Briggs AM Rowbotham S Schütze R O'Sullivan PB Smith AJ Tory B Stinson JN Lord SM Slater H. “It's kind of just like a never-ending cycle”: young people's experiences of co-existing chronic musculoskeletal pain and mental health conditions. J Pain 2025;32:105412. 40316036 10.1016/j.jpain.2025.105412

[21] Klem N-R Slater H Rowbotham S Chua J Waller R Stinson JN Romero L Lord SM Tory B Schütze R Briggs AM. Lived and care experiences of young people with chronic musculoskeletal pain and mental health conditions: a systematic review with qualitative evidence synthesis. PAIN 2025;166:732–54. 39445766 10.1097/j.pain.0000000000003407 PMC11921448

[22] Long AC Palermo TM. Brief report: web-based management of adolescent chronic pain: development and usability testing of an online family cognitive behavioral therapy program. J Pediatr Psychol 2009;34:511–6. 18669578 10.1093/jpepsy/jsn082 PMC2684485

[23] Mohabir V Lalloo C Eccleston C Hess C Pham Q Slater H Velmovitsky P Stinson J. The transformative potential of digital therapeutics in pediatric pain. PAIN 2025;166:S121–30. 41086343 10.1097/j.pain.0000000000003713

[24] Murray CB Groenewald CB de la Vega R Palermo TM. Long-term impact of adolescent chronic pain on young adult educational, vocational, and social outcomes. PAIN 2020;161:439–45. 31651579 10.1097/j.pain.0000000000001732 PMC7001863

[25] Murray CB de la Vega R Murphy LK Kashikar-Zuck S Palermo TM. The prevalence of chronic pain in young adults: a systematic review and meta-analysis. PAIN 2022;163:e972–84. 34817439 10.1097/j.pain.0000000000002541

[26] Nagata JM Imbago-Jácome D Choonara S Talebloo J Memon Z O'Sullivan M Sawyer SM Baird S, Lancet Youth Commissioners. Reporting of research with adolescent and youth engagement. Lancet Child Adolesc Health 2025;9:442–5. 40409336 10.1016/S2352-4642(25)00092-6

[27] Rigby E Hagell A Davis M Gleeson H Mathews G Turner G. Getting health services right for 16-25 year-olds. Arch Dis Child 2021;106:9–13. 32561543 10.1136/archdischild-2019-318648

[28] Scott IA van der Vegt A Lane P McPhail S Magrabi F. Achieving large-scale clinician adoption of AI-enabled decision support. BMJ Health Care Inform 2024;31:e100971. 10.1136/bmjhci-2023-100971 PMC11141172 38816209

[29] Slater H Briggs AM. Strengthening the pain care ecosystem to support equitable, person-centered, high-value musculoskeletal pain care. PAIN 2024;165:S92–107. 39560420 10.1097/j.pain.0000000000003373

[30] Slater H Jordan JE Chua J Schütze R Wark JD Briggs AM. Young people's experiences of persistent musculoskeletal pain, needs, gaps and perceptions about the role of digital technologies to support their co-care: a qualitative study. BMJ Open 2016;6:e014007. 10.1136/bmjopen-2016-014007 PMC5168607 27940635

[31] Slater H Stinson JN Jordan JE Chua J Low B Lalloo C Pham Q Cafazzo JA Briggs AM. Evaluation of digital technologies tailored to support young people's self-management of musculoskeletal pain: mixed methods study. J Med Int Res 2020;22:e18315. 10.2196/18315 PMC7305555 32442143

[32] Slater H Waller R Briggs AM Lord SM Smith AJ. Characterizing phenotypes and clinical and health utilization associations of young people with chronic pain: latent class analysis using the electronic Persistent Pain Outcomes Collaboration database. PAIN 2025;166:67–86. 39688968 10.1097/j.pain.0000000000003326 PMC11647817

[33] Stinson JN Lalloo C Harris L Isaac L Campbell F Brown S Ruskin D Gordon A Galonski M Pink LR Buckley N Henry JL White M Karim A. iCanCope with Pain^TM^: user-centred design of a web- and mobile-based self-management program for youth with chronic pain based on identified health care needs. Pain Res Manag 2014;19:257–65. 25000507 10.1155/2014/935278 PMC4197753

[34] Thabrew H Fleming T Hetrick S Merry S. Co-design of eHealth interventions with children and young people. Front Psychiatry 2018;9:481. 30405450 10.3389/fpsyt.2018.00481 PMC6200840

[35] Tory B. The pain of being a young person experiencing chronic pain. Lancet Child Adolesc Health 2024;8:257–8.

[36] Treede R-D Rief W Barke A Aziz Q Bennett MI Benoliel R Cohen M Evers S Finnerup NB First MB Giamberardino MA Kaasa S Korwisi B Kosek E Lavand’homme P Nicholas M Perrot S Scholz J Schug S Smith BH Svensson P Vlaeyen JWS Wang S-J. Chronic pain as a symptom or a disease: the IASP classification of chronic pain for the International Classification of Diseases (ICD-11). PAIN 2019;160:19–27. 30586067 10.1097/j.pain.0000000000001384

[37] van der Vegt A Campbell V Zuccon G. Why clinical artificial intelligence is (almost) non-existent in Australian hospitals and how to fix it. Med J Aust 2024;220:172–5. 38146620 10.5694/mja2.52195

[38] Vargas C Whelan J Brimblecombe J Allender S. Co-creation, co-design, co-production for public health—a perspective on definitions and distinctions. Public Health Res Pract 2022;32:e3222211. 10.17061/phrp3222211 35702744

[39] Voorheis P Petch J Pham Q Kuluski K. Maximizing the value of patient and public involvement in the digital health co-design process: a qualitative descriptive study with design leaders and patient-public partners. PLoS Digital Health 2023;2:e0000213. 37878566 10.1371/journal.pdig.0000213 PMC10599516

[40] World Health Organization. Youth-centred digital health interventions: A framework for planning, developing and implementing solutions with and for young people, 2021. Available at: https://www.who.int/publications/i/item/9789240011717. Accessed September 8, 2025.

[41] World Health Organization. WHO framework for meaningful engagement of people living with noncommunicable diseases, and mental health and neurological conditions, 2023. Available at: https://www.who.int/publications/i/item/9789240073074. Accessed September 8, 2025.

